# Successful Operation for the Cure of Laryngismus Paralyticus or Roaring the Horse

**Published:** 1889-10

**Authors:** J. S. Butler

**Affiliations:** Piqua, Ohio


### ART. XX.—SUCCESSFUL OPERATION FOR THE CURE OF LARYNGISMUS PARALYTICUS OR ROARING IN THE HORSE.

BY J. S. BUTLER, V.S.,
*Piqua, Ohio.*

AT the request of Messrs. Dye and Stilwell, importers and breeders of English Shire horses, Troy, Ohio, Dr. T. J. Pence of that place secured a roarer and wished me to operate on him as an experiment. Never having operated on such a case, I thought best to invite several veterinarians to be present to assist and witness the operation.

The 12th day of June was fixed for the operation, the following veterinarians being present: Drs. Howe, Shaw, Charlesworth, Labron, Logan, Kerr, Derr, Pence and the writer.

I was assisted in the operation by Drs. Howe and Charlesworth, Drs. Kerr and Logan administering the anesthetic.

The subject was a gelding at least twenty years old, and in an emaciated condition. He had a chronic discharge from the nasal passages with considerable rattling in those passages and trachea. The least exertion would cause very loud roaring, and upon being severely exerted would stagger and nearly fall unless stopped.

The readers of this article will no doubt agree with the writer that this was not a very favorable subject for such an operation. I had never seen the subject till the day he was operated upon.

The animal was thrown and secured, and a suitable stage of anesthesia was reached in about ten minutes. The hair was clipped as closely as possible over the region of the larynx, the skin was sponged with a solution of hydrarg-bichloride, then an incision about five inches in length was made in the median line through the skin and muscles, exposing the larynx and trachea. The hemorrhage was controlled by cold water sponging and torsion of some vessels.

When the hemorrhage had entirely ceased the larynx was opened together with the first two rings of the trachea. Considerable mucus mixed with pus escaped from the wound coming up from the trachea and bronchi.

The true cause of roaring in this case was fully demonstrated as the left arytenoid cartilage was perfectly immovable, and the muscles of the same side were plainly atrophied, while the right arytemoid and muscles were in a normal condition and expanded and contracted at each respiratory act.

The left vocal cord was severed from its anterior attachment, and it with the arytemoid cartilage was entirely removed. All partially detached pieces of tissue and shreds of mucous membrane were taken away by the means of curved shears. As the hemorrhage was slight it was not thought necessary to pack the trachea. What hemorrhages ensued was controlled by cold water autiseptically treated with hydrarg-bichlor., applied with small silk sponges attached to the end of pieces of whalebone.

The wound was left open as advised by Dr. Fleming and the operation throughout was performed as nearly as possible by his method.

After the animal had sufficiently recovered from the anesthetic he was allowed to arise and walk back to the barn.

A thin piece of gause was tied around his neck covering the wound, and kept wet with the antiseptic solution, and a little of the solution was injected in the wound.

No food or water was allowed for thirty-six hours, when a few swallows of water and a little bran and oatmeal gruel was given him which he swallowed without much difficulty.

The wound was dressed antiseptically twice a day, and the diet was confined to gruel for several days when a little grass was allowed.

On the 9th day after the operation Dr. Pence and I threw the animal again and examined the interior of the larynx and wound ; we found it in good condition except in two or three places the granulations were rather profuse. These were touched with a solution of argenti nitras, and continued the former treatment for four weeks, when the wound was about healed.

Then a run of seventy-five yards was given him without producing any roaring.

A few days afterwards the wound having healed, the animal was turned out on grass. The former owner one day went to the pasture without our knowledge, got on his back and galloped him all over the pasture, giving him a severe test, but without producing any roaring.

On July 24th, about six weeks after the operation, Drs. Pence, Charlesworth and the writer went to the pasture to give him the final test, we put a halter on him and ran him up hill and down, and in every other way we could, but without producing any roaring.

The respirations were somewhat harsh, which was unquestionably due to chronic thickening of the nasal and tracheal mucous membranes.

